# Renal cell carcinoma with a tumor thrombus in the ureter: a case report

**DOI:** 10.1186/1471-2490-11-16

**Published:** 2011-08-01

**Authors:** Osamu Fujita, Koichiro Wada, Tomoya Yamasaki, Daisuke Manabe, Katsuji Takeda, Satoko Nakamura

**Affiliations:** 1 Department of Urology, Kagawa Prefectural Central Hospital, 5-4-16, Ban-cho, Takamatsu, Kagawa, 760-8557, Japan; 2 Department of Pathology, Kagawa Prefectural Central Hospital, 5-4-16, Ban-cho, Takamatsu, Kagawa, 760-8557, Japan

## Abstract

**Background:**

Renal cell carcinoma (RCCs) is the most common malignancy of the kidney. When RCC progresses, it is known to form tumor thrombus in the renal vein and/or inferior vena cava. However, RCC does not normally form tumor thrombus in the ureter or renal pelvis.

**Case presentation:**

A 43-year-old man presented to our department for the treatment of a renal tumor with asymptomatic gross hematuria. In a dynamic CT study, contrast enhancement revealed a tumor suspected to be RCC, but atypical finding as a tumor thrombus that filled the renal pelvis and the whole ureter was also observed. Nephroureterectomy was performed, and the tumor was diagnosed histopathologically as RCC.

**Conclusion:**

We report here a very rare case of RCC with a tumor thrombus in the whole ureter.

## Background

Renal cell carcinoma (RCCs) is the most common malignancy arising from the kidney. While local renal tumor growth and extension may involve the perirenal fat, adrenal glands, renal vein, inferior vena cava, urinary collecting system and/or adjacent retroperitoneal structure, RCC normally form tumor thrombus neither in the renal pelvis nor ureter.

Urinary Collecting System Invasion (UCSI) of RCC including tumor thrombus in the renal pelvis and/or ureter has not been classified in the UICC TNM classification because of less frequency [1]. The case in the present report is very rare, RCC with formation of a tumor thrombus that directly extended into the renal pelvis and filled the whole ureter.

## Case presentation

**Figure 1:**
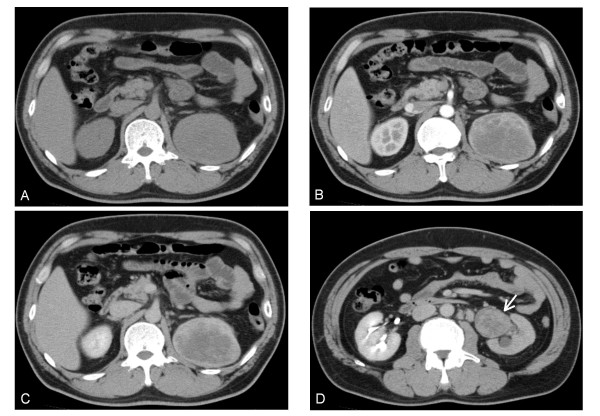
**Horizontal section of preoperative abdominal CT**. Plain (A), early-phase contrast enhancement (B), and late phase (C) showed contrast enhancement. A tumor thrombus in the left renal pelvis (D, arrow), and its contrast enhancement was similar to the main tumor.

**Figure 2:**
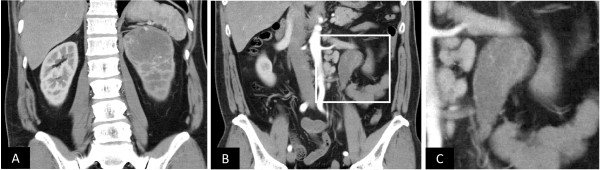
**Coronal section of preoperative abdominal CT**. A solid tumor in the upper pole of the left kidney (A) and the tumor thrombus (B) showed early-phase contrast enhancement.

**Figure 3:**
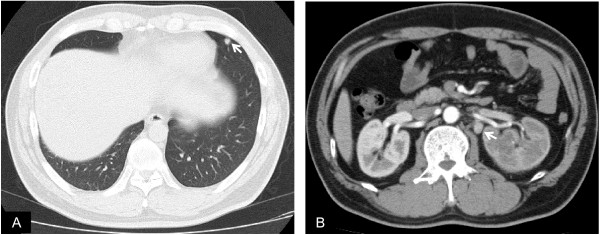
**Preoperative CT images showed a nodule in the left lung (A, arrow), and para-aortic lymph node enlargement (B, arrow)**.

A 43-year-old man who had a history of right renal calculus presented to a nearby hospital with a chief complaint of asymptomatic gross hematuria. A left renal tumor was suspected based on plain abdominal CT, and he was referred to our department. Physical examination revealed no abnormality in the chest, the abdomen or the extremities. Laboratory data on admission revealed mild leukocytosis, with leucocyte of 10.5 × 10^3^/μL, mild hypochromic anemia, with hemoglobin of 12.9 g/dL, and mild inflammatory reaction, with erythrocyte sedimentation rate (ESR) of 24 mm at the first hour, C-reactive protein (CRP) of 4.0 mg/dL. Urinalysis revealed erythrocyte count of 50-99/HPF and no pyuria or bacteriuria. Urine cytology was class I and showed no atypical cells. A dynamic CT study revealed a tumor (8 × 7 cm in diameter) in the upper pole of the left kidney (Figures 1A) and which showed contrast enhancement in the early phase (Figures 1B, C and 2A) and extended into the left renal pelvis and the ureter (Figures 1D). There was also contrast enhancement at this site (Figures 2B and 2C). In addition, a nodule with 8 mm in diameter was found in the S5 segment of the left lung (Figure 3A), and para-aortic lymph nodes enlargement were observed (Figure 3B). Cystoscopy revealed no obvious tumorous lesion. The tumor was diagnosed as left RCC (cT3aN2M1), and transabdominal left nephrectomy and hilar lymph node dissection were performed. Also total ureterectomy including cuff resection of bladder wall was additionally performed because intraoperative histopathological examination couldn't rule out urothelial carcinoma.

**Figure 4:**
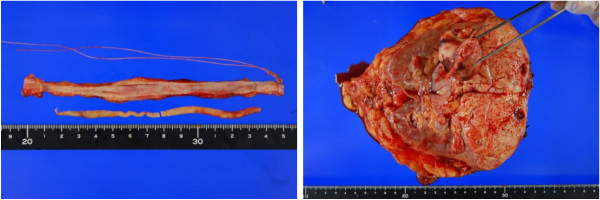
**The macroscopic findings of the long tumor thrombus**.

**Figure 5:**
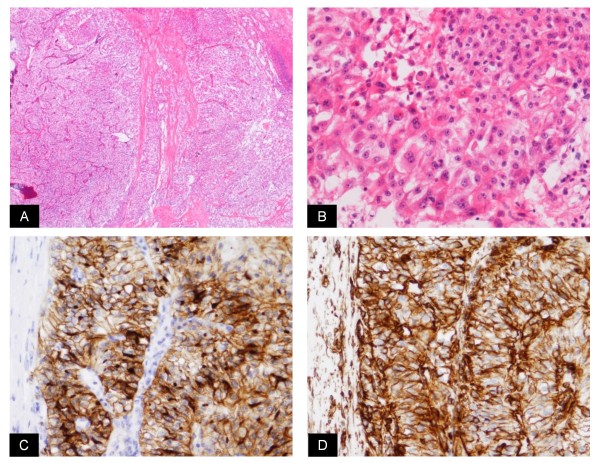
**Histological findings (A; HE, ×40, B; HE, ×200)**. Immunostaining was positive for CD10 and vimetin (C, D; ×100).

Histopathological examination: The macroscopic findings of the excised tumor were a yellow color and no hemorrhage was observed in its interior. There was a protrusion into the renal sinus fat, renal pelvis and a cord-shaped tumor thrombus of approximately 17 cm extending to the lower ureter (Figure 4). Histologically, atypical epithelium with severe necrosis, invasion and proliferation of these atypical cells were observed. In all areas of the renal tumor but lymph nodes, cytoplasm of tumor cells contained glycogen (Figure 5A and 5B). Immunostaining was positive for CD10 and vimetin (Figure 5C and 5D). According to these findings, the tumor was diagnosed as clear cell RCC (G2 > G3 >> G1, INFβ, v(+), pT3apN0). No malignancy was observed in the surrounding renal pelvic or ureteral mucosa.

## Discussion

The case in the present report had RCC with tumor thrombus formation in the renal pelvis and whole ureter. Based on preoperative examinations including imaging procedures, we had difficulty to differentiate it from renal pelvic cancer and collecting duct renal cell carcinoma. Furthermore, the tumor was difficult to differentiate by intraoperative histopathological examination. Thus, this case required careful consideration regarding surgery. Finally nephroureterectomy was performed, including resection of the tumor thrombus in the ureter. The patient has had no local recurrence to date.

In 2007, Gulati et al. reported the only other case of RCC that directly invaded from the renal pelvis into the ureter and formed a tumor thrombus [2]. A tumor thrombus is thought to advance into the vein due to pressure in the vein and the renal pelvis. Gulati et al. reported that venous pressure was increased due to the renal vein tumor thrombus and lymph node compression [2]. In the patient in the present report, there was microscopic venous invasion but not tumor thrombus in the renal vein, and no massive lymph node enlargement/metastasis that was large enough to increase the venous pressure. Therefore, the tumor thrombus was thought to have spontaneously spread directly into the ureter by UCSI.

Some articles have reported about UCSI [3-7]. According to those reports, UCSI of RCC is not so rare (7.5-14%), but Uzzo et al reported that no case of local involvement of the ureter by tumor was identified in 61 cases with UCSI [3]. It is controversial that whether UCSI should be added into TNM classification [6,7], however most reported UCSI of RCC to be a significant predictor of prognosis [3,5-7]. Thus, the case in the present report is very rare and must be followed up carefully.

## Conclusions

We report here a very rare case of RCC with a tumor thrombus in the whole ureter.

## Consent

Written informed consent was obtained from the patients for publication of this case report and accompanying images. A copy of the written consent is available for review by the Editor-in-Chief of this journal.

## List of abbreviations used

UICC: Union for International Cancer Control; HPF: High Power Field; CD10: Cluster of Differentiation 10

## Competing interests

The authors declare that they have no competing interests.

## Authors' contributions

OF drafted the first manuscript. KW, TY, DM, and KT cared for the patients. SN performed histological examinations and pathological investigation. KW, TY, DM, and KT also helped to draft the manuscript. All authors reviewed the report and approved the final version of the manuscript.

## Pre-publication history

The pre-publication history for this paper can be accessed here:

http://www.biomedcentral.com/1471-2490/11/16/prepub
